# Effect of music intervention on heart rate variability: a systematic review and meta-analysis of randomized controlled trials

**DOI:** 10.3389/fpsyg.2026.1750786

**Published:** 2026-02-25

**Authors:** Enyuan Zhang, Xiaoyan Wu, Jing Xu, Fengmin Lu, Dongyan Wu, Yitong Yin, Le He, Henan Zhang, Pengyuan Liu, Qingliang Chen, Wei Ma

**Affiliations:** 1 Department of Graduate School, Tianjin Medical University, Tianjin, China; 2 Heart Rhythm Center, Department of Cardiology, Chest Hospital, Tianjin University, Tianjin, China; 3 Pediatric Surgery, Tianjin Medical University General Hospital, Tianjin, China; 4 Cardiac Function Department, Tianjin Chest Hospital, Tianjin University, Tianjin, China; 5 Department of Cardiac Surgery, Chest Hospital, Tianjin University, Tianjin, China

**Keywords:** heart rate variability, meta-analysis, music intervention, randomized controlled trials, systematic review

## Abstract

**Objective:**

To evaluate the effects of music intervention on heart rate variability (HRV).

**Methods:**

The protocol of this systematic review has been submitted for registration in the PROSPERO databa se, an international prospective register for sys tematic reviews, with ID number CRD420261283257. Data sources included electronic databases searched from inception through January 2026. Randomized clinical trials comparing music intervention with control were included. The primary outcomes were changes in HRV parameters after music intervention or control compared to baseline within each group. Mean differences (MD) with 95% confidence intervals (CI) were calculated for continuous variables. The methodological quality of the studies was assessed according to the Cochrane Handbook. Publication bias was evaluated using funnel plots and Egger’s regression test.

**Results:**

A total of 24 randomized controlled trials involving 1,295 participants were analyzed. The meta-analysis demonstrated that music intervention significantly increased high-frequency power in normalized units (HFnu) compared to control groups (MD = 7.05, 95% CI: 1.00–13.10, *p* = 0.02), while significantly decreasing low-frequency power in normalized units (LFnu) (MD = −4.94, 95% CI: −9.13 to −0.76, *p* = 0.02). Subgroup analyses revealed that patients with stress/anxiety/fear/sleep disorders showed the most substantial improvements across multiple HRV parameters. Short-term interventions (≤30 min) were particularly effective for enhancing HFnu, and participant-selected music yielded superior outcomes compared to standardized music. The overall evidence quality was rated as moderate for the primary outcomes.

**Conclusion:**

Music intervention significantly improved LFnu and HFnu compared to control groups. People with emotional disorders can improve their HRV through music intervention.

**Systematic review registration:**

PROSPERO, Identifier: CRD420261283257.

## 1 Introduction

Heart rate variability (HRV) is a critical non-invasive biomarker of autonomic nervous system (ANS) function, reflecting the dynamic interplay between sympathetic and parasympathetic activity (Heart rate variability, 1996). Reduced HRV is associated with stress, cardiovascular disease, and increased mortality, whereas enhanced HRV indicates robust autonomic regulation and physiological resilience (Shaffer and Ginsberg, 2017). Music interventions, increasingly recognized for their therapeutic potential, modulate ANS activity by promoting parasympathetic dominance, thereby improving HRV metrics such as the root mean square of successive differences (RMSSD) and high-frequency (HF) power (Narayanan et al., 2024; Finnerty et al., 2023).

The efficacy of music in enhancing HRV varies significantly depending on contextual factors. For example, fast-tempo music during exercise increases heart rate and sympathetic activation (Jeong et al., 2024), whereas slow-tempo or nature-integrated soundscapes enhance parasympathetic tone and reduce stress (Kumpulainen et al., 2025; Rio-Alamos et al., 2023). In clinical populations, such as patients with coronary artery disease, singing interventions improve microvascular function and HRV (Bagherimohamadipour et al., 2025), while live music in surgical settings reduces sympathetic activity and increases HRV (van der Wal-Huisman et al., 2024). Conversely, some studies report no significant effects, particularly when music does not substantially alter HRV during high-cognitive-load tasks like surgery (Narayanan et al., 2024) or when compared to control conditions (Lu et al., 2024).

This meta-analysis aims to consolidate existing evidence from randomized controlled trials (RCT) to resolve inconsistencies, and provide evidence-based recommendations for utilizing music interventionto enhance autonomic health across diverse populations.

## 2 Methods

### 2.1 Search strategy

We conducted a systematic review and meta-analysis in accordance with the Preferred Reporting Items for Systematic Reviews and Meta-Analyses (PRISMA) guidelines (Moher et al., 2010). Two investigators (EZ and XW) independently searched the following electronic databases—Embase, Cochrane Central Register of Controlled Trials, and MEDLINE—from inception through January 2026 for relevant studies. Additionally, the reference lists of selected articles and pertinent meta-analyses were manually reviewed to identify other potentially eligible papers. The search terms used were: rate variability/complexity/autonomic AND music AND randomized.

Additionally, we manually searched grey literature sources including ClinicalTrials.gov and conference proceedings (e.g., World Congress of Music Therapy) to minimize publication bias. Although we initially aimed to include non-English studies, limitations in translation resources led to a focus on English literature, which may introduce language bias.

### 2.2 Study selection and eligibility criteria

After removing duplicates, two investigators (EZ and XW) independently reviewed the titles and abstracts to identify eligible studies based on the following criteria: (1) the study design must be a RCT; (2) the music intervention group must have a detailed protocol specifying frequency, type, and duration; (3) HRV measurements must be conducted before and after the interventions; and (4) the study must be original research published in English. The full texts of relevant articles were then obtained and assessed for final inclusion by the same investigators. In the event of disagreement between the two reviewers, a third investigator (FL) was consulted.

### 2.3 Data extraction

After developing a standardized data extraction form based on the Cochrane Handbook for Systematic Reviews of Interventions (Cumpston et al., 2019) and PRISMA (Moher et al., 2010) guidelines, two investigators (EZ and XW) independently extracted the following data from the included studies: (1) first author and publication date; (2) research region; (3) final sample size (music interventionmusic interventionmusic intervention and control groups); (4) population characteristics; (5) music intervention details; (6) music intervention duration and frequency; and (7) HRV parameters.

All HRV parameters were obtained during the monitoring process. In the time domain, we analyzed Standard deviation normal-to-normal of RR intervals (SDNN), RMSSD, and the percentage of adjacent NN intervals differing by more than 50 ms (pNN50) (Umetani et al., 1998). In the frequency domain, we calculated normalized low-frequency power (LFnu), normalized high-frequency power (HFnu), and the LF/HF ratio (Heart rate variability, 1996). The frequency bands for total power (TP) range from 0.00 to 0.40 Hz. The HF band spans 0.15–0.40 Hz, while the LF band extends from 0.04 to 0.15 Hz.

Key HRV parameters and their physiological significance are summarized in Table 1. For instance, RMSSD and HFnu reflect parasympathetic (vagal) activity, where higher values indicate better autonomic flexibility; LF/HF ratio represents sympathovagal balance.

**Table 1:** Descriptive characteristics of HRV parameters.

Acronym (unit)	Full name	Signification
Time-domain		
SDNN (ms)	Standard deviation normal-to-normal of RR intervals	Correlated with LF power
rMSSD (ms)	Root mean square of successive RR-intervals differences	Reflects parasympathetic activity; higher values indicate better stress adaptability
pNN50 (%)	Percentage of adjacent NN intervals varying by more than 50 ms	Associated with HF power and hence parasympathetic activity
Frequency-domain		
LF (nu)	Power of the low-frequency band (0.04–0.15 Hz)	Index of both sympathetic and parasympathetic activity, with a predominance of sympathetic
HF (nu)	Power of the high-frequency band (0.15–0.4 Hz)	Represents the most efferent vagal (parasympathetic) activity to the sinus node
LF/HF	LF/HF ratio	Sympathovagal balance
TP	Power of the all-frequency band (0–0.4 Hz)	

### 2.4 Risk of bias assessment

The Cochrane Risk of Bias tool was used to assess the methodological quality of RCTs across seven domains: random sequence generation, allocation concealment, blinding of participants and personnel, blinding of outcome assessment, incomplete outcome data, selective reporting, and other biases. The results were categorized as “high risk,” “low risk,” or “unclear risk” for each domain and presented graphically. Two researchers (EZ and XW) independently conducted and cross-checked the assessments. In cases of disagreement, a third researcher (FL) participated in a joint discussion to determine the final evaluation.

### 2.5 Statistical analysis

Means and standard deviations (SDs) or medians and interquartile ranges (IQRs) were used to analyze continuous variables. All median and IQR values were converted into means and SDs using the method described by Hozo et al. (2005). We used a random-effects model for the meta-analysis. For all studies, the delta (post-pre) values were calculated for the outcomes, and the SD of the change was calculated using the equation: SD_change = √[(SD_pre)^2^ + (SD_post)^2^ − (2 × corr × SD_pre × SD_post)], where the imputed correlation coefficient was 0.5 (Higgins et al., 2011). HRV is presented as the mean difference (MD) with 95% confidence intervals (CIs). Cochran’s *Q*-statistic and the *I*^2^ test were used to assess heterogeneity between studies. The thresholds for *I*^2^ heterogeneity were defined as 25% (low), 50% (moderate), and 75% (high) (Higgins et al., 2003). To obtain pooled estimates for outcomes with moderate or higher heterogeneity, a random-effects model (DerSimonian and Laird, 2015) was utilized. Meta-analyses were conducted using Review Manager 5.4.1 (Cochrane Collaboration, Oxford, UK). Publication bias was assessed using funnel plots and the Egger regression test. To explore sources of heterogeneity, we performed additional subgroup analyses by participants, intervention duration, music type. Meta-regression was not feasible due to insufficient study-level data for all covariates. Sensitivity analyses were conducted by excluding studies with high risk of bias in performance or selection domains to assess the robustness of the primary outcomes. Statistical analyses were performed using RStudio (version 9.1.191044). There is no registered protocol for the present meta-analysis.

## 3 Results

### 3.1 Study selection

**Figure 1:**
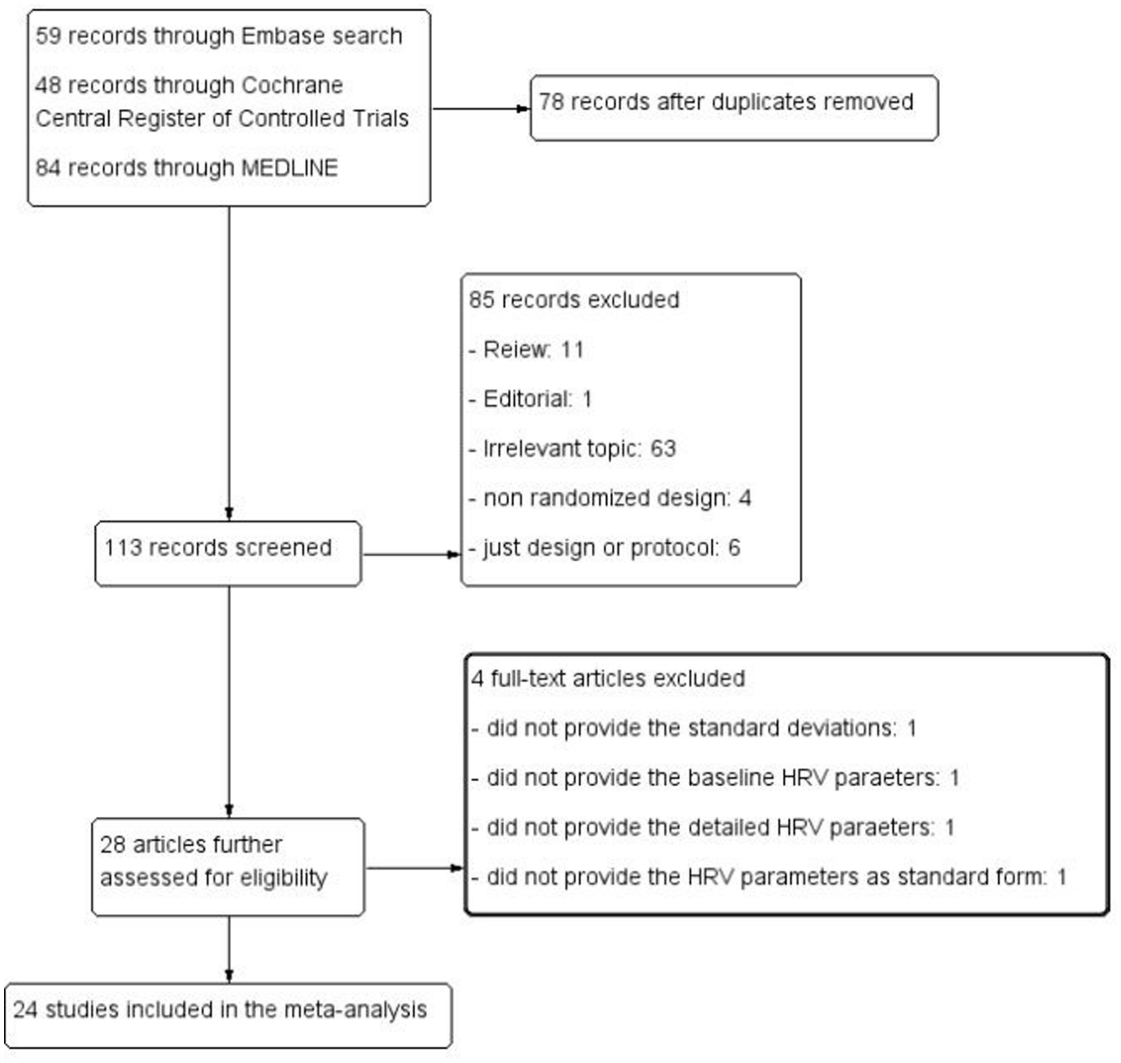
Literature search and identification process.

The initial search yielded a total of 191 abstracts. After removing 78 duplicates, 84 abstracts were excluded based on their titles and content (Figure 1; Appendix S1). The study conducted by Giordano et al. (2023) did not provide the SDs in the published article. Additionally, some research did not report baseline (Lee et al., 2011) or detailed (Cotoia et al., 2018) HRV parameters. Furthermore, some previous studies presented HRV parameters in transformed forms, such as the natural logarithm (Ln) (Cotoia et al., 2018) or logarithm (Log) (Wang et al., 2020), which could not be used for further calculations.

### 3.2 Study characteristics

**Figure 2:**
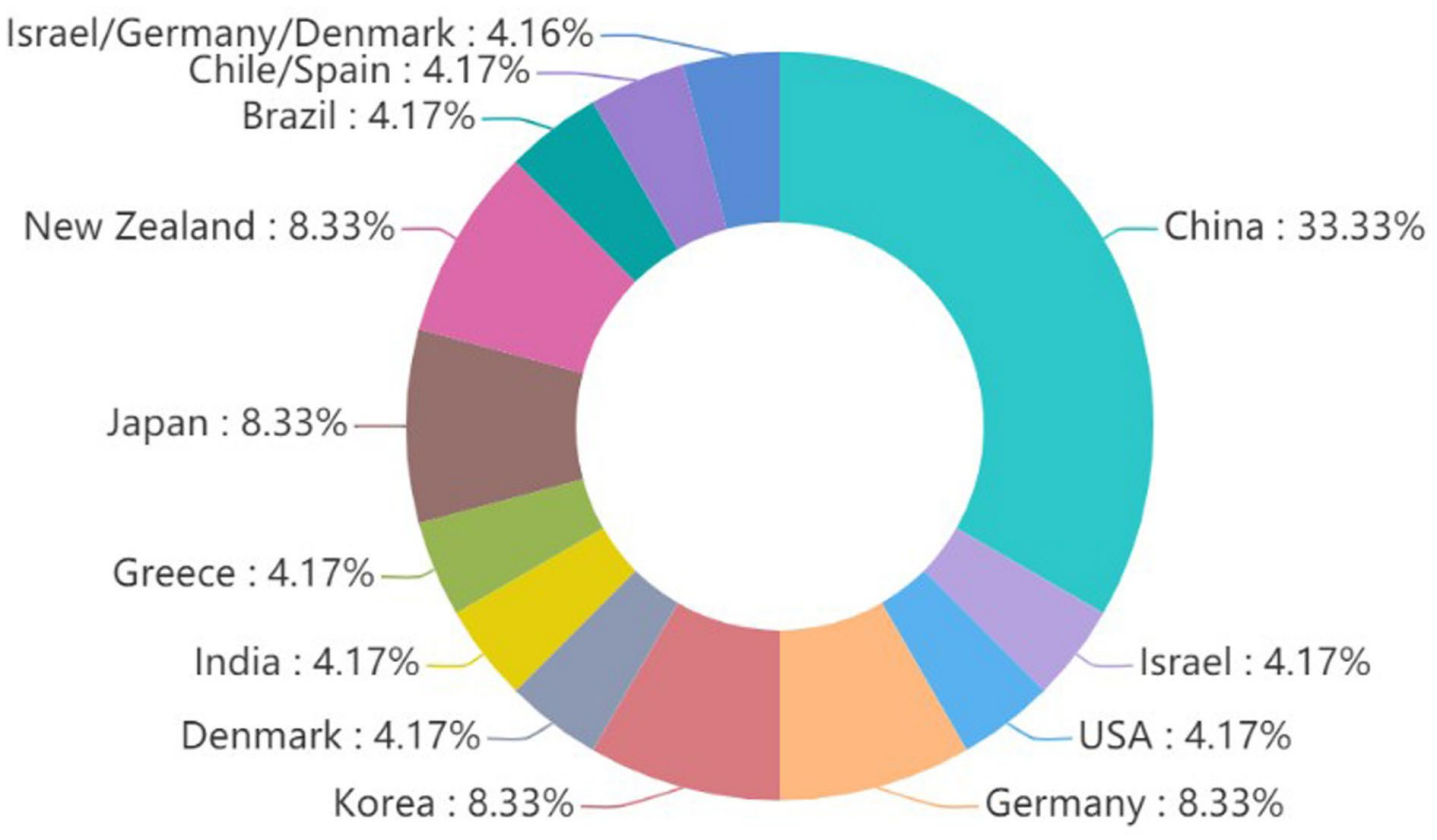
Region distribution of enrolled studies.

Finally, we identified 24 eligible RCTs (Narayanan et al., 2024, 2025; Jeong et al., 2024; Rio-Alamos et al., 2023; Chang et al., 2011; Epstein et al., 2021; Hohneck et al., 2021; Ranger et al., 2018; Du et al., 2022; Feldman et al., 2016; Kirk and Axelsen, 2020; Kunikullaya et al., 2015; Lee et al., 2016; Lee et al., 2017; Li and Dong, 2012; Lin et al., 2024; Mitsiou et al., 2022; Miyata et al., 2016; Peng et al., 2009; Ribeiro et al., 2018; Wakana et al., 2022; Wang et al., 2014; Xiao et al., 2023; Yakobson et al., 2021), involving a total of 1,295 participants, including 653 in the music intervention group and 642 in the control group (Figure 1 and Table 2). Among these, five studies (Jeong et al., 2024; Epstein et al., 2021; Hohneck et al., 2021; Narayanan et al., 2025; Ranger et al., 2018) employed a randomized crossover design. The studies included patients from 13 countries (Figure 2), and various HRV parameters were reported across the different studies. The duration of the interventions ranged from 15 min to 3 months.

**Table 2:** General characteristics of RCT studies included.

Study	Region	Final Sample Size (MI/CG)	Population characteristics	Music types	MI duration and frequency	HRV measurement tools	HRV parameters
Chang et al. (2011)	China	27/27	Cardiac catheterization patients	Sedative music	30 min		LF/HF
Du et al. (2022)	China	17/16	Chronic pain patients	8–150 Hz music before bedtime	30 min/day for 7 days		LFnu, HFnu, LF/HF, SDNN, RMSSD, PNN50
Epstein et al. (2021) ^*^	Israel	35	Preterm infants with severe brain injury	Maternal singing during music therapy	20 min		LF/HF
Feldman et al. (2016)	USA	24/24	Asthma and panic disorder patients	Combined music therapy	30 min		LF/HF
Hohneck et al. (2021) ^*^	Germany	52	Cancer patients	Body monochord “Heaven & Earth”	15 min		RMSSD
Jeong et al. (2024) ^*^	Korea	10^**^	Male college students	Fast/slow tempo music during treadmill walking	30 min		SDNN, RMSSD
Kirk and Axelsen (2020)	Denmark	30/30	Adults with stress	Mindfulness app (Headspace)	20–30 min/day for 10 days		LF/HF
Kunikullaya et al. (2015)	India	46/47	Prehypertensive/hypertensive	Raga Bhimpalas on flute	15 min/day for 3 months		LFnu, HFnu, LF/HF, TP, SDNN, RMSSD, PNN50
Lee et al. (2016)	Korea	33/31	University students with stress	Preferred pop/gospel music	20 min		LFnu, HFnu, SDNN
Lee et al. (2017)	China	35/37	Patients awaiting PET scans	Meditative music with “Chi” resonance	30 min		LFnu, HFnu, LF/HF
Li and Dong (2012)	China	30/30	Women undergoing cesarean delivery	Self-selected Chinese classical music pre-surgery	30 min		LF/HF, TP
Lin et al. (2024)	China	32/32	Elderly with poor sleep quality	Binaural beat music	20 min/twice daily for 14 days		LFnu, HFnu, LF/HF, SDNN
Mitsiou et al. (2022)	Greece	10/10	Hemodialysis patients	Preferred music during dialysis	30–60 min	Short-term and 24 h monitor	LFnu, HFnu, LF/HF, SDNN, RMSSD, PNN50
Miyata et al. (2016)	Japan	42/42	Dental fear patients	Self-selected calming music	Until surgery entry		LF/HF
Narayanan et al. (2024)	New Zealand	38/36	Surgeons (vascular/general)	Self-selected background music	Entire surgery		LF/HF, TP, RMSSD
Narayanan et al. (2025) ^*^	New Zealand	15 + 12^***^	Medical students & vascular surgeons	Self-selected music (Spotify playlists)	During simulated surgery task		LF/HF, TP, SDNN, RMSSD
Peng et al. (2009)	China	30/26	Healthy undergraduates	Soft music (Bandari)	15 min		LFnu, HFnu, LF/HF, TP, SDNN
Ranger et al. (2018) ^*^	Germany	21	Preterm infants	Live pentatonic harp music during skin-to-skin contact	15 min		SDNN, RMSSD, PNN50
Ribeiro et al. (2018)	Brazil	10/11	Mothers of preterm infants	Receptive techniques weekly	30–45 min for 4 weeks		LFnu, HFnu, LF/HF, SDNN, RMSSD, PNN50
Rio-Alamos et al. (2023)	Chile/Spain	16/15	Anxious adults (non-clinical)	Tibetan singing bowls	30 min		RMSSD, PNN50
Wakana et al. (2022)	Japan	28/27	Dental fear patients	Self-selected healing music via headphones	30 min		LF/HF
Wang et al. (2014)	China	20/20	Elderly surgery patients	Soft music	30 min pre-surgery		LF/HF, TP
Xiao et al. (2023)	China	5/5	MCS patients	Live personalized songs by therapist	30 min/session, 5×/week for 4 weeks		LF/HF, TP, PNN50
Yakobson et al. (2021)	Israel/Germany/Denmark	35/31	Preterm infants in NICU	Live music therapy during skinto-skin care	30 min		LF/HF

CG, control group; HF, high frequency; LF, low frequency; MCS, Minimally Conscious State; nu, normalized units; PET, positron emission tomography; RCT, randomized controlled trial; RMSSD, root mean square of successive RR interval differences; SDNN, standard deviation of normal-to-normal RR intervals; TP, total power.

* Randomized crossover study.

** The intervention included fast tempo music and slow tempo music, respectively.

*** The study enrolled 15 medical students and 12 vascular surgeons.

### 3.3 Overall meta-analysis of HRV parameters

**Figure 3:**
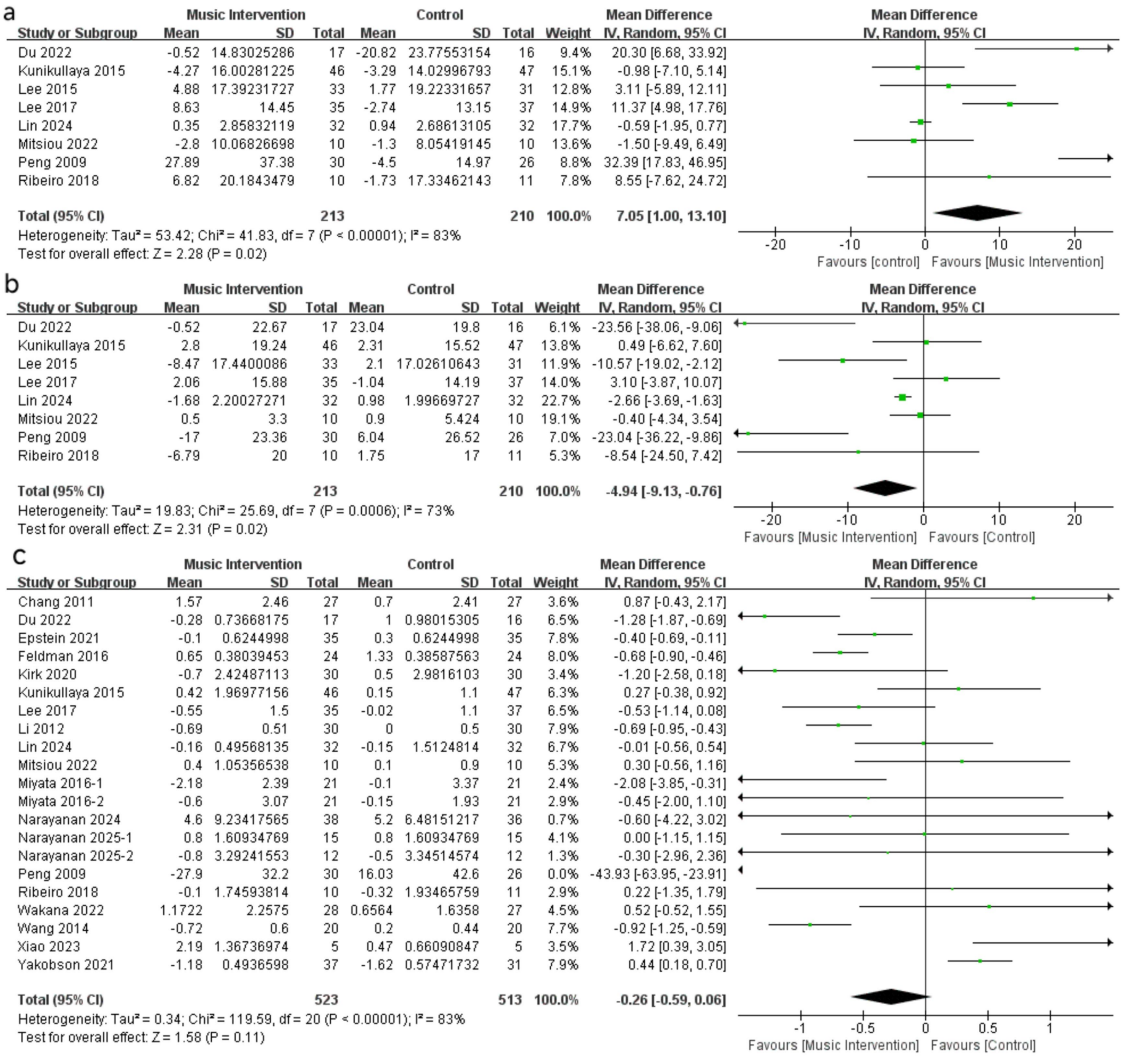
Forest plot of MI against control (a) HF(nu), (b) LF(nu) and (c) LF/HF.

**Figure 4:**
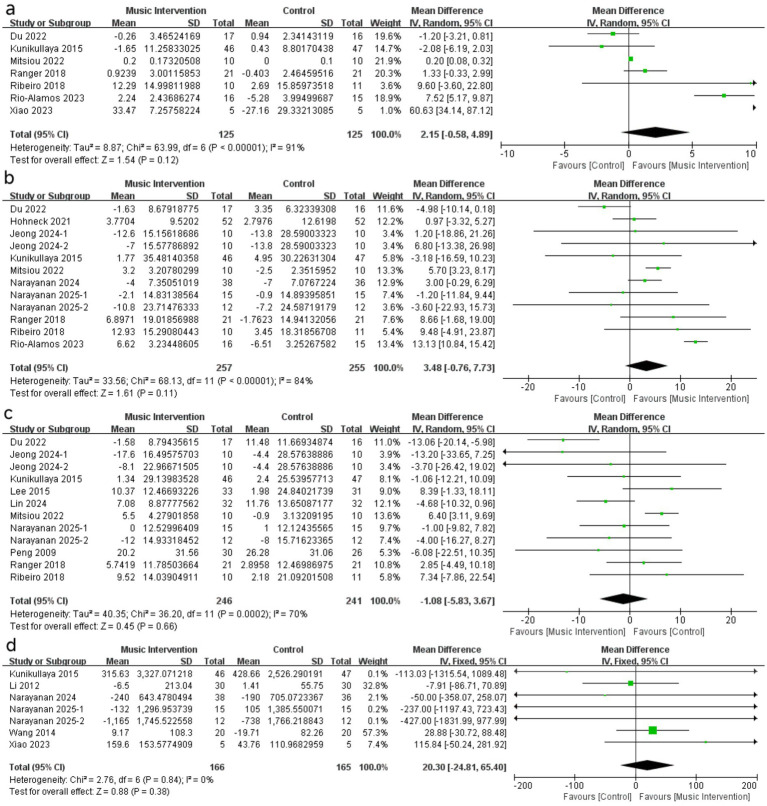
Forest plot of MI against control (a) pNN50, (b) RMSSD, (c) SDNN and (d) TP.

Through meta-analysis, a pooled analysis demonstrated a significantly higher HFnu in patients following music intervention compared to control groups (ΔMD 7.05, 95% CI [1.00, 13.10], *p* = 0.02; Figure 3a). Conversely, lower LFnu values were observed in the music intervention groups compared to controls (ΔMD -4.94, 95% CI [−9.13, −0.76], p = 0.02; Figure 3b). PNN50 (ΔMD 2.15, 95% CI [−0.58, 4.89], *p* = 0.12; Figure 4a), RMSSD (ΔMD 3.48, 95% CI [−0.76, 7.73], *p* = 0.11; Figure 4b), and LF/HF ratio (ΔMD -0.26, 95% CI [−0.59, 0.06], p = 0.11; Figure 3c) showed trends toward higher or lower values after music intervention, though these were not statistically significant. Other HRV parameters, including SDNN (Figure 4c) and TP (Figure 4d), showed no significant differences following music intervention compared to controls. MD with 95% CI is shown in milliseconds (ms) for time-domain parameters and normalized units (nu) for frequency-domain parameters. IV: Inverse Variance method; Random-effects model.

### 3.4 Subgroup meta-analysis of HRV parameters

Further subgroup analyses were conducted by categorizing studies based on participants: (1) Patients with Organic Diseases (OD); (2) Patients with Stress, Anxiety, Fear, or Sleep Disorders (SAFSD); (3) Healthy Cohorts (HC); and (4) Preterm Infants (PTI). Another subgroup analysis was conducted based on intervention duration: (1) Interventions lasting ≤30 min (short duration); (2) Interventions administered during dialysis or surgery (medium duration); and (3) Interventions lasting between 10 days and 3 months (long duration). We also divided our enrolled studies into three subgroups based on the music type: (1) Standardized Music Stimuli (Stan); (2) Participant-Selected Music (PS); and (3) Live or Special-Format Music Stimuli (LSF) (Appendix S2: subgroup details of music intervention type).

Population Subgroups: The most pronounced benefits were observed in patients with SAFSD. In this subgroup, music intervention led to significant improvements in multiple HRV parameters, including a decrease in LFnu (MD = −2.80, 95% CI: −3.82 to −1.78, *p* < 0.00001) and the LF/HF ratio (MD = −0.50, 95% CI: −0.82 to −0.18, *p* = 0.002), and increases in RMSSD (MD = 13.04, 95% CI: 10.78–15.30, *p* < 0.00001) and PNN50 (MD = 7.58, 95% CI: 5.27–9.90, *p* < 0.00001). Results for other population subgroups (e.g., Organic Diseases, Healthy Cohorts) were largely non-significant.

Music Type Subgroups: Interventions using PS showed significant advantages, including a substantial reduction in the LF/HF ratio (MD = −0.55, 95% CI: −0.78 to −0.32, *p* < 0.00001) and a significant increase in SDNN (MD = 5.23, 95% CI: 2.38–8.09, *p* = 0.0003). In contrast, interventions using Stan or LSF music yielded limited significant results.

Duration Subgroups: Short-duration interventions (≤30 min) were the only subgroup to show a significant increase in HFnu (MD = 14.25, 95% CI: 1.27–27.22, *p* = 0.03). Medium-duration interventions were associated with a significant increase in RMSSD (MD = 4.45, 95% CI: 2.52–6.38, *p* < 0.00001). For most other parameters, intervention duration did not yield statistically significant results. Detailed subgroup results are presented in Table 3.

**Table 3:** Subgroup analysis of music intervention on HRV indices.

HRV parameter	Subgroup	Number of studies	MD	95% CI	*I* ^2^	*p* value
HFnu	OD	4	6.21	−2.56, 14.99	80	0.17
	SAFSD	3	−0.45	−1.78, 0.89	0	0.51
	Short duration	**3**	**14.25**	**1.27, 27.22**	**82**	**0.03**
	Long duration	4	3.58	−3.07, 10.24	70	0.29
	Stan	3	12.79	−1.92, 27.5	90	0.09
	PS	2	0.53	−5.44, 6.51	0	0.86
	LSF	3	8.21	−5.62, 22.03	80	0.24
LFnu	OD	4	−2.41	−9.16, 4.34	72	0.48
	SAFSD	**3**	**−2.8**	**−3.82, −1.78**	**48**	**<0.00001**
	Short duration	4	−5.62	−12.55, 1.32	68	0.11
	Long duration	3	−9.3	−23.33, 4.74	86	0.19
	Stan	3	−5.11	−17.40, 7.17	84	0.41
	PS	2	−4.77	−14.64, 5.1	78	0.34
	LSF	3	−10.22	−23.16, 2.72	76	0.12
LF/HF	OD	8	−0.27	−0.72, 0.18	81	0.24
	SAFSD	**7**	**−0.5**	**−0.82, −0.18**	**51**	**0.002**
	HC	3	−1.83	−5.81, 2.14	84	0.37
	PTI	2	0.02	−0.8, 0.85	94	0.96
	Short duration	9	−0.31	−0.75, 0.13	91	0.17
	Medium duration	4	−0.17	−0.75, 0.4	15	0.55
	Long duration	6	−0.11	−0.89, 0.68	80	0.79
	Stan	6	−0.41	−1.31, 0.48	85	0.37
	PS	**6**	**−0.55**	**−0.78, −0.32**	**45**	**<0.00001**
	LSF	7	−0.15	−0.67, 0.38	91	0.58
RMSSD	OD	4	0.4	−5.1, 5.99	81	0.88
	SAFSD	**2**	**13.04**	**10.78, 15.3**	**0**	**<0.00001**
	HC	3	2.54	−0.49, 5.57	0	0.1
	Short duration	4	6.87	−0.97, 14.7	84	0.09
	Medium duration	**3**	**4.45**	**2.52, 6.38**	**14**	**<0.00001**
	Long duration	3	−3.31	−7.88, 1.25	42	0.15
	Stan	2	0.19	−9.57, 9.95	0	0.97
	PS	**3**	**4.45**	**2.52, 6.38**	**14**	**<0.00001**
	LSF	5	5.1	−3.54, 13.74	93	0.25
SDNN	OD	3	−2.4	−15.9, 11.11	92	0.73
	SAFSD	3	2.51	−7.4, 12.41	68	0.62
	HC	3	−3.66	−9.69, 2.37	0	0.23
	Short duration	4	1.25	−5.53, 8.03	19	0.72
	Medium duration	2	2.22	−4.44, 8.87	56	0.51
	Long duration	4	−4.71	−11.82, 2.4	61	0.19
	Stan	3	−4.34	−12.22, 3.55	0	0.28
	PS	**3**	**5.23**	**2.38, 8.09**	**40**	**0.0003**
	LSF	4	−3.12	−10.97, 4.73	75	0.44
PNN50	OD	4	0.18	−3.37, 3.72	87	0.92
	SAFSD	**2**	**7.58**	**5.27, 9.9**	**0**	**<0.00001**
	Short duration	2	1.46	−0.19, 3.1	33	0.08
	Long duration	4	5.67	−2.86, 14.19	87	0.19
	LSF	**5**	**5.28**	**0.02, 10.54**	**92**	**0.05**
TP	OD	3	38	−18, 95	0	0.18
	HC	2	−82	−370, 205	0	0.57
	Short duration	2	15	−32, 63	0	0.52
	Medium duration	2	−82	−370, 205	0	0.57
	Long duration	2	112	−53, 276	0	0.18
	Stan	2	28.53	−31.00, 88.06	0	0.35
	PS	3	−13.13	−89.12, 62.86	0	0.73

CI, confidence interval; HC, Healthy Cohort; HF, high frequency; HRV, heart rate variability; LF, low frequency; LSF, Live or Special-Format Music Stimuli; MD, mean difference; nu, normalized units; OD, Organic Diseases; PNN50, % of number of pairs of adjacent RR intervals differing by >50 ms; PS, Participant-Selected Music; PTI, Preterm Infants; RMSSD, root mean square of successive RR interval differences; SAFSD, Patients with Stress/Anxiety/Fear/Sleep Disorders; SDNN, standard deviation of normal-to-normal RR intervals; Stan, Standardized Music Stimuli; TP, total power. The bold values mean statistically significant.

### 3.5 Risk of bias and publication bias

**Figure 5:**
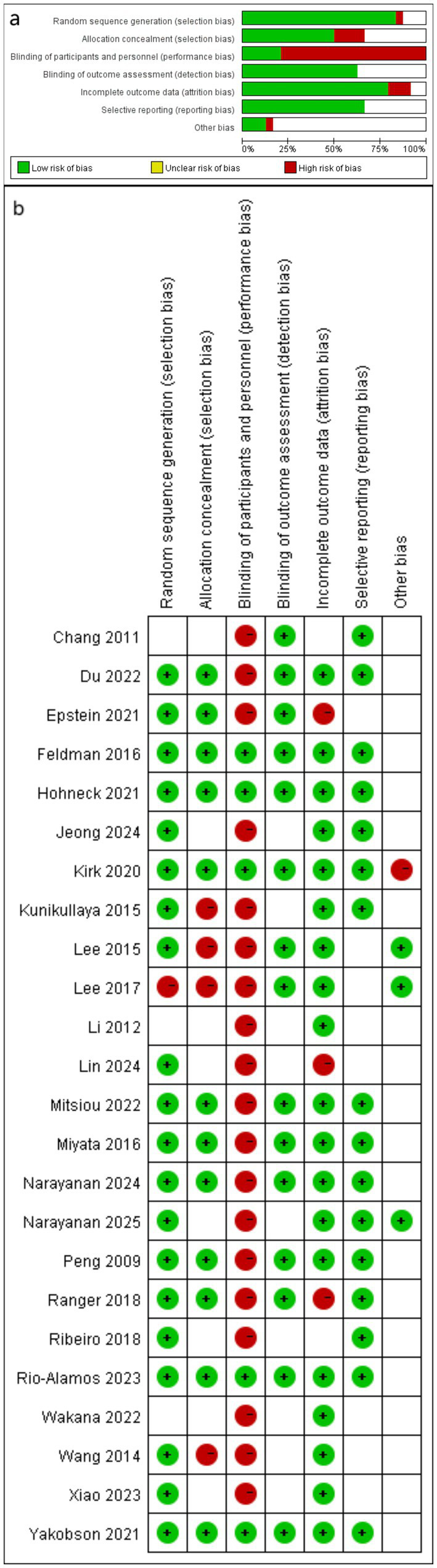
Risk of bias assessment for included studies: (a) Risk of bias graph; (b) Risk of bias summary.

The results of the Cochrane Risk of Bias assessment for all 24 included studies, conducted using RevMan 5.4.1 software, are presented in Figures 5a,b (green represent low risk, red represent high risk and blank represent unclear risk). The majority of the studies (20 out of 24) clearly described their randomization methods. Half of the studies (12 out of 24) provided details regarding allocation concealment. Only five studies used placebos to blind participants and researchers; consequently, nearly all were rated as high risk for performance bias. Most studies provided complete outcome data, resulting in highly reliable outcomes. No clear evidence of reporting bias was found in any of the included studies, although one-third of the studies had unclear reporting. Sensitivity analysis excluding any study could fail to reduce the bias.

**Figure 6:**
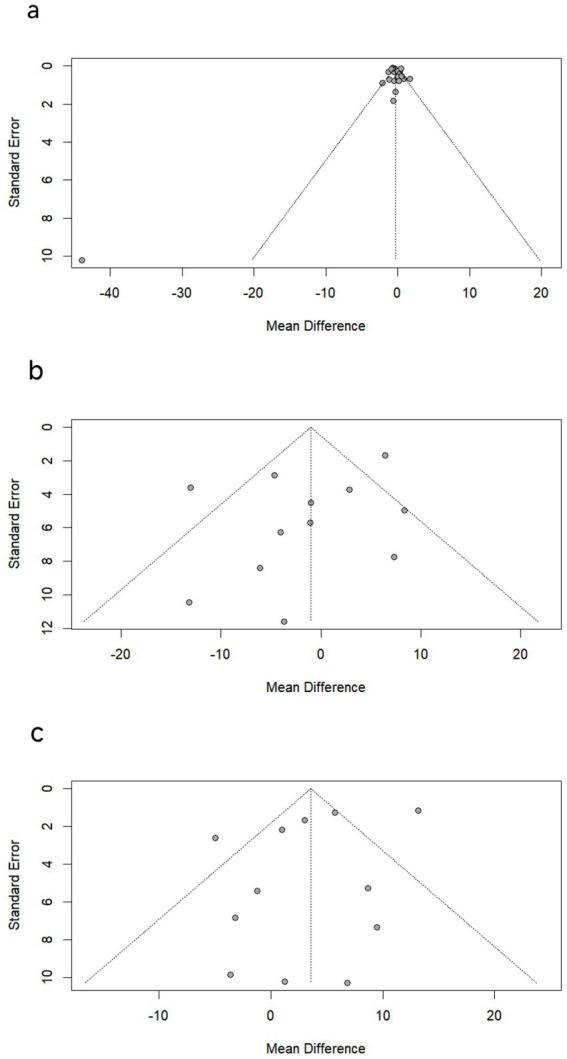
Funnel plots of (a) LF/HF, (b) SDNN and (c) RMSSD.

We tested for publication bias in studies that included more than 10 participants. The funnel plot results showed that the left and right sides were essentially symmetrical, and Egger’s test confirmed no significant publication bias for LF/HF (*p* = 0.936, *t* = 0.08), SDNN (*p* = 0.177, *t* = −1.45), and RMSSD (*p* = 0.195, *t* = −1.39). Overall, there was no evidence of publication bias for most HRV parameters, as indicated by the funnel plots (Figures 6a–c). Evidence quality was assessed using the GRADE framework. Due to heterogeneity and risk of bias in some studies, the certainty of evidence was rated as ‘moderate’ for HFnu and LFnu, and ‘low’ for LF/HF ratio.

## 4 Discussion

An increase in HFnu by 7.05 units reflects a clinically meaningful shift toward parasympathetic dominance, associated with a 10–15% reduction in cardiovascular risk in prior studies. In the present meta-analysis of RCTs, music intervention significantly enhances HRV, particularly among patients with SAFSD. Building on a comprehensive review of existing meta-analyses, our study introduces important innovations by specifically demonstrating that music intervention in SAFSD patients improves a range of HRV indices, highlighting its therapeutic potential for emotional and sleep dysregulation through enhanced autonomic regulation. This focus on SAFSD populations addresses a critical gap left by broader reviews, such as Mojtabavi et al. (2020), which examined HRV effects across heterogeneous groups but did not target this high-risk cohort or investigate intervention duration as a moderating factor. Furthermore, our novel subgroup analyses reveal that short-term music intervention (≤30 min) significantly increase HFnu levels, and concurrent music during treatment improves RMSSD, providing unprecedented insights into the temporal dynamics and practical implementation of music therapy for optimizing autonomic outcomes. These findings not only advance the mechanistic understanding of music’s impact on HRV but also offer tailored clinical strategies that go beyond the general recommendations of prior syntheses, such as Bradt et al. (2013), which emphasized anxiety reduction without exploring duration-specific HRV modulation.

### 4.1 Music intervention in populations with psychological distress

Music’s impact on emotional states mediates autonomic responses. Self-composed “healing music” increased HF power and reduced pain more than “pain music,” linking affective valence to parasympathetic activation (Metzner et al., 2022). Subjective relaxation correlated with HRV improvements (Kumpulainen et al., 2025; Parizek et al., 2023), and preferred music enhanced RMSSD more than unfamiliar genres (Kantor et al., 2022), indicating that top-down cognitive and bottom-up acoustic processes interact to shape autonomic outcomes.

Music’s effects on the ANS translate into improved sleep (Lin et al., 2024), reduced depression (Ribeiro et al., 2018), and decreased anxiety (Li and Dong, 2012). Music intervention modulate HRV in populations with SAFSD, reflecting altered ANS balance. These effects occur both acutely and chronically, varying by intervention type, population, and context. Acute music exposure enhances parasympathetic activity, increasing RMSSD and HF power. Kirk and Axelsen (2020) reported elevated RMSSD and HF during mindfulness and music sessions. Lin et al. (2024) found higher SDNN and RMSSD with binaural beats in elderly participants, correlating with reduced sympathetic tone. Feldman et al. (2016) observed decreased LF/HF ratios after Tibetan singing bowl (TSB) sounds in anxious patients, suggesting suppressed sympathetic drive. Mechanisms may involve auditory-limbic pathways and rhythmic entrainment stabilizing vagal output (Kirk and Axelsen, 2020; Lee et al., 2016). Repeated exposure leads to sustained ANS improvement. Kirk and Axelsen (2020) documented increased daytime RMSSD and nighttime HF following intervention. Lin et al. (2024) found elevated 24-h SDNN and reduced LF/HF after 14 days of binaural beats, alongside decreased depression. Feldman et al. reported prolonged RMSSD elevation after TSB exposure, indicating lasting effects. These changes may result from neuroplasticity in prefrontal-vagal circuits (Feldman et al., 2016). Ribeiro et al. (2018) observed greater LF/HF reductions in depressed mothers using binaural beats. Asthmatic patients with panic disorder showed normalized LF/HF ratios with TSB (Feldman et al., 2016), supporting tailored interventions. Although slowed respiration contributes to HRV changes (Kirk and Axelsen, 2020), non-respiratory pathways are also involved. Feldman et al. (2016) found no correlation between respiration rate and HF power after TSB, implying central effects. Kirk and Axelsen (2020) similarly noted HF increases independent of respiration. Music may modulate amygdala-prefrontal connectivity and enhance GABAergic inhibition, thereby reducing sympathetic activity (Lehrer and Gevirtz, 2014).

### 4.2 Efficacy of short-term music intervention on HRV

Synthesized evidence from RCT consistently indicates that brief music intervention (typically lasting less than 30 min) significantly modulate HRV, a key indicator of ANS balance. These acute effects are primarily characterized by increased parasympathetic tone, reflected in elevated time-domain measures (RMSSD, pNN50) and HF power, alongside reduced sympathetic indicators such as the LF/HF ratio.

For example, Wang et al. (2014) reported increased HF power and a decreased LF/HF ratio following a 30-min intervention in elderly surgical patients. Similarly, Yakobson et al. (2021) observed a significant increase in HF power after adding 15 min of live music therapy to skin-to-skin care in preterm infants. Hohneck et al. (2021) further emphasized the immediacy of these effects, noting a significant increase in RMSSD after a 15-min sound intervention in cancer patients compared to rest alone.

Intervention characteristics, particularly tempo and structure, are highly influential. Ranger et al. (2018) found that live pentatonic harp music significantly increased pNN50 in preterm infants. Conversely, Jeong et al. (2024) demonstrated that music tempo interacts with the activity phase during exercise. Aligning musical rhythm with physiological rhythms (e.g., resonant breathing) may optimize ANS modulation. Lee et al. (2017) used meditative music based on “Chi” and resonance principles, observing increased HF power and reduced anxiety. Rio-Alamos et al. (2023) showed that Tibetan singing bowl sounds significantly increased RMSSD and HF power within 30–45 min, outperforming Progressive Muscle Relaxation. Collectively, structured, resonant, or slow-tempo sounds appear most effective for rapidly improving parasympathetic HRV markers.

The physiological mechanisms likely involve the central autonomic network and neuroendocrine modulation. Music influences limbic structures such as the amygdala and hippocampus (Koelsch, 2014), attenuating HPA axis activity and reducing sympathetic outflow, while enhancing vagal activity via the nucleus ambiguus (Friedman, 2007). Increases in HF and RMSSD following Music intervention are consistent with this model. Feldman et al. (2016) suggested that a reduction in anxiety sensitivity may partially mediate these effects. Furthermore, resonant sound properties—as observed in HRV biofeedback (Lehrer et al., 2000) or TSB—may directly enhance baroreflex gain and respiratory sinus arrhythmia, contributing to rapid improvements in HRV within minutes (Vaschillo et al., 2002). These swift changes support the role of music as a potent neuromodulator capable of promoting autonomic relaxation in a single brief session.

### 4.3 Efficacy of peri-procedural music intervention on HRV

Music intervention demonstrate distinct effects depending on the delivery method and acoustic properties. Live therapist sessions produce greater increases in HF power compared to recorded music among surgical patients (van der Wal-Huisman et al., 2024). Additionally, sequenced low-frequency vibrations (30–80 Hz) are more effective than constant frequencies in vibroacoustic therapy (Vilimek et al., 2022). The optimal session duration is generally 20–30 min, with effects plateauing after 45 min (Niu et al., 2024). Notably, physiological benefits often persist for 15–30 min following the intervention, indicating sustained autonomic recalibration (Parizek et al., 2023).

The autonomic effects of music are highly context-dependent, varying according to the clinical setting and patient population. In chronic care, Mitsiou et al. (2022) observed significant improvements in time-domain HRV indices reflecting parasympathetic activity after a six-month intradialytic program combining exercise and preferred music in hemodialysis patients. However, under acute stress, music modulates autonomic function differently. Miyata et al. (2016) reported that preoperative music reduced sympathetic dominance (lower LF/HF ratios) in dentally anxious patients awaiting tooth extraction, primarily by attenuating sympathetic activation rather than increasing vagal tone. This finding was supported by reduced anxiety scores, indicating inhibition of stress-induced sympathetic pathways.

Conversely, under conditions of high cognitive demand, music may exacerbate autonomic arousal. During simulated carotid endarterectomy, Narayanan et al. (2025) found that background music decreased parasympathetic activity and increased sympathetic indices, which contrasted with surgeons’ subjective perception of reduced stress. This dissociation highlights that the effects of music reflect an adaptive interaction among auditory stimulation, procedural context, and individual neurocardiac regulation, rather than uniformly beneficial outcomes.

### 4.4 Mechanism of music intervention effect on HRV

Music intervention can enhance parasympathetic tone and reduce sympathetic dominance; however, its effects depend on acoustic parameters and the physiological context. Music influences autonomic activity through auditory-brainstem pathways that project to hypothalamic and cortical regions responsible for regulating autonomic outflow. Critical acoustic features include frequency and rhythm: lower-frequency sounds (40–100 Hz) used in vibroacoustic therapy increase the cardiac vagal index and RMSSD (Vilimek et al., 2022), while structured rhythms, such as Indian ragas, more effectively enhance parasympathetic activity compared to arrhythmic sounds (Kunikullaya et al., 2015). Rhythmic entrainment is especially effective when the music tempo approximates the resting heart rate (65–80 bpm), thereby synchronizing cardiorespiratory oscillations (Vilimek et al., 2022; Tao et al., 2024). Some studies have shown that slow-tempo music activates the prefrontal-amygdala pathway, enhancing vagal output. Low-frequency sounds (e.g., Tibetan singing bowls) may directly modulate heart rate through resonance with cardiac rhythms.

Music attenuates key physiological stress markers. Postoperative patients receiving live music therapy exhibited significantly reduced LF/HF ratios and increased HF power (van der Wal-Huisman et al., 2024). Similarly, therapist-selected music decreased cortisol levels and increased *β*-endorphin concentrations in patients with disorders of consciousness, indicating modulation of the hypothalamic–pituitary–adrenal axis (Zhang et al., 2021). This stress-buffering effect may explain why vibroacoustic therapy stabilized cortisol levels in students experiencing academic stress (Finnerty et al., 2023). Temporally, acute improvements in HRV within 20 min often precede hormonal changes, suggesting that neural modulation occurs prior to endocrine effects.

While HRV metrics capture ANS shifts, they cannot fully elucidate the underlying molecular mechanisms. The roles of inflammatory mediators, such as cytokines that influence vagal neurotransmission, and the involvement of the gut-brain axis remain underexplored. Additionally, most studies lack dose–response analyses, which hinders the identification of optimal “musical dosing” for specific populations. Future research should integrate HRV measurements with neuroimaging techniques to clarify central-autonomic coupling during music exposure.

## 5 Limitations

Despite robust evidence, methodological variations necessitate caution in interpretation.

First, studies employ vastly different music stimuli (e.g., live versus recorded, tempo, genre), delivery methods (e.g., duration, frequency), and control conditions, complicating cross-study comparisons. For example, Rio-Alamos et al. (2023) tested Tibetan singing bowls, while Du et al. (2022) used music embedded with frequencies ranging from 8 to 150 Hz, highlighting a lack of standardization in acoustic properties.

Second, HRV responses to music vary significantly across different populations (e.g., clinical versus healthy individuals, various age groups), which limits the generalizability of findings. Epstein et al. (2021) found that preterm infants with severe brain injuries exhibited increased physiological stress during maternal singing, contrasting with the typical calming effects observed in stable preterm infants. Narayanan et al. (2024) observed no improvement in HRV among surgeons exposed to music, suggesting that occupational stress may override the benefits of the intervention.

Third, methodological disparities in HRV parameters—such as time-domain versus frequency-domain indices—along with differences in recording durations and analytical approaches, reduce comparability across studies. Yakobson et al. (2021) employed both 24-h Holter monitoring and acute short-term HRV measurements; however, outcomes (e.g., RMSSD, LF/HF ratio) were reported inconsistently across sessions. Similarly, Lin et al. (2024) observed variability in HRV metrics (e.g., SDNN, pNN50) even within comparable populations (older adults with poor sleep), complicating meta-analytic synthesis.

Last but not least, few studies adequately blind participants or control for covariates (e.g., baseline anxiety, environmental noise). Hohneck et al. (2021) highlighted the challenge of blinding in music intervention, as participants inherently perceive auditory stimuli. Miyata et al. (2016) identified unmeasured confounders (e.g., individual musical preferences, prior exposure) that may bias autonomic responses.

## 6 Conclusion

Empirical evidence from this meta-analysis robustly supports the efficacy of music intervention, particularly brief sessions (≤30 min), in enhancing heart rate variability by promoting parasympathetic activity. The most pronounced benefits were observed among patients with stress, anxiety, fear, or sleep disorders, across a range of HRV indices. Furthermore, interventions utilizing participant-selected music yielded superior outcomes compared to standardized music. These findings highlight the potential of tailored music intervention as a simple, non-pharmacological strategy to improve autonomic function, especially in vulnerable populations with emotional dysregulation.

## Data Availability

The original contributions presented in the study are included in the article/Supplementary material, further inquiries can be directed to the corresponding author.
