# Supplementary material for: Effect of music intervention on heart rate variability: a systematic review and meta-analysis of randomized controlled trials
Source: Front Psychol. 2026 Feb 25;17:1750786. doi: 10.3389/fpsyg.2026.1750786 (PMC12976007; doi:10.3389/fpsyg.2026.1750786)
Supplement: Supplementary file 1 [file Supplementary_file_1.docx]

A comprehensive literature search was performed to identify all relevant randomized controlled trials (RCTs) investigating the effect of music intervention on heart rate variability (HRV). The search was conducted across three major electronic databases: Embase, the Cochrane Central Register of Controlled Trials (CENTRAL), and MEDLINE (via PubMed). The search strategy utilized a combination of Medical Subject Headings (MeSH) terms and free-text keywords related to "music," "heart rate variability," "autonomic nervous system," and "randomized controlled trial." The search was not restricted by publication date or language.

The initial database searches yielded a total of 191 citations (59 from Embase, 48 from CENTRAL, and 84 from MEDLINE). After removing 113 duplicate records, 78 unique records remained for screening. The titles and abstracts of these records were screened against the predefined eligibility criteria, leading to the exclusion of 85 records. The primary reasons for exclusion at this stage were: being a review article (n=11), an editorial (n=1), irrelevant topic (n=63), or having a non-randomized study design (n=4). Subsequently, the full texts of the remaining 28 articles were thoroughly assessed for eligibility. Of these, 4 full-text articles were excluded for the following reasons: failure to provide standard deviations of outcomes (n=1), lack of baseline HRV parameters (n=1), absence of detailed HRV parameter data (n=1), and not reporting HRV parameters in a standard form (n=1).

Consequently, 24 studies met all inclusion criteria and were included in the final quantitative synthesis (meta-analysis). The study selection process is detailed in the PRISMA flow diagram
