# Supplementary material for: Effect of music intervention on heart rate variability: a systematic review and meta-analysis of randomized controlled trials
Source: Front Psychol. 2026 Feb 25;17:1750786. doi: 10.3389/fpsyg.2026.1750786 (PMC12976007; doi:10.3389/fpsyg.2026.1750786)
Supplement: Supplementary file 2 [file Supplementary_file_2.docx]

Based on the "Music types" column in the provided table, the studies can be classified into the following three groups. The grouping rationale is based on the nature and source of the music stimulus used in the intervention.

Grouping Rationale

1. Standardized Music Stimuli: This group includes studies where the music was a pre-selected, standardized recording or a specific type of music chosen by the researchers. The stimulus is consistent across all participants and focuses on the acoustic properties of the music itself (e.g., sedative, soft, a specific Raga).

2. Participant-Selected Music: This group includes studies where the music was chosen by the participants based on their personal preference. The key element here is the individualization of the stimulus to match the listener's taste.

3. Live or Special-Format Music Stimuli: This group includes interventions that involve live music performance, music therapy techniques, or specially engineered sounds (e.g., binaural beats, specific frequencies). The focus is on the interactive, therapeutic, or unique physical properties of the sound intervention beyond standard music listening.

Grouping Results

Group 1: Standardized Music Stimuli

• Chang 2011: Sedative music

• Jeong 2024*: Fast/slow tempo music during treadmill walking (Tempo controlled by researchers)

• Kunikullaya 2015: Raga Bhimpalas on flute (Specific, researcher-selected Raga)

• Lee 2017: Meditative music with "Chi" resonance (Researcher-selected type)

• Peng 2009: Soft music (Bandari) (Researcher-selected artist/genre)

• Wang 2014: Soft music (Researcher-selected type)

• Kirk 2020: Mindfulness app (Headspace) (Standardized audio content from an app)

Group 2: Participant-Selected Music

• Lee 2015: Preferred pop/gospel music

• Li 2012: Self-selected Chinese classical music pre-surgery

• Mitsiou 2022: Preferred music during dialysis

• Miyata 2016: Self-selected calming music

• Narayanan 2024: Self-selected background music

• Narayanan 2025*: Self-selected music (Spotify playlists)

• Wakana 2022: Self-selected healing music via headphones

Group 3: Live or Special-Format Music Stimuli

• Du 2022: 8-150Hz music before bedtime (Music with specific frequency parameters)

• Epstein 2021*: Maternal singing during music therapy (Live, therapeutic singing)

• Feldman 2016: Combined music therapy (Music therapy techniques)

• Hohneck 2021*: Body monochord "Heaven & Earth" (Music therapy instrument)

• Ranger 2018*: Live pentatonic harp music during skin-to-skin contact (Live music performance)

• Ribeiro 2018: Receptive techniques weekly (Music therapy techniques)

• Rio-Alamos 2023: Tibetan singing bowls (Sound from a special instrument)

• Xiao 2023: Live personalized songs by therapist (Live, interactive music therapy)

• Yakobson 2021: Live music therapy during skin-to-skin care (Live music therapy)

• Lin 2024: Binaural beat music (Engineered auditory illusion)

* Randomized crossover study
